# Development of New Models of Oral Mucosa to Investigate the Impact of the Structure of Transmembrane Mucin-1 on the Mucosal Pellicle Formation and Its Physicochemical Properties

**DOI:** 10.3390/biomedicines12010139

**Published:** 2024-01-09

**Authors:** Clément Nivet, Irma Custovic, Laure Avoscan, Floris J. Bikker, Aline Bonnotte, Eric Bourillot, Loïc Briand, Hélène Brignot, Jean-Marie Heydel, Noémie Herrmann, Mélanie Lelièvre, Eric Lesniewska, Fabrice Neiers, Olivier Piétrement, Mathieu Schwartz, Christine Belloir, Francis Canon

**Affiliations:** 1 Center for Taste and Feeding Behaviour (CSGA), UMR1324 INRAE, Institut Agro Dijon, Université de Bourgogne, UMR6265 CNRS, 21000 Dijon, France; clement.nivet@inrae.fr (C.N.); loic.briand@inrae.fr (L.B.); helene.brignot@inrae.fr (H.B.); jean-marie.heydel@u-bourgogne.fr (J.-M.H.); noemie.herrmann@inrae.fr (N.H.); melanie.lelievre@inrae.fr (M.L.); fabrice.neiers@u-bourgogne.fr (F.N.); mathieu.schwartz@inrae.fr (M.S.); christine.belloir@inrae.fr (C.B.); 2 Institut Carnot de Bourgogne (ICB), UMR CNRS 6303, University of Bourgogne, 21000 Dijon, France; irma.custovic@u-bourgogne.fr (I.C.); eric.bourillot@u-bourgogne.fr (E.B.); eric.lesniewska@u-bourgogne.fr (E.L.); olivier.pietrement@u-bourgogne.fr (O.P.); 3 Agroécologie, UMR1347 INRAE, ERL CNRS 6300, DimaCell Platform, Center of Microscopy INRAE, University of Bourgogne, 21000 Dijon, France; laure.avoscan@inrae.fr (L.A.); aline.bonnotte@inrae.fr (A.B.); 4 Department of Oral Biochemistry, Academic Centre for Dentistry Amsterdam, University of Amsterdam and VU University Amsterdam, 1081 LA Amsterdam, The Netherlands; f.bikker@acta.nl

**Keywords:** MUC1, mucosal pellicle, MUC5B, salivary protein interactions, oral mucosa, AFM-IR

## Abstract

The mucosal pellicle (MP) is a biological film protecting the oral mucosa. It is composed of bounded salivary proteins and transmembrane mucin MUC1 expressed by oral epithelial cells. Previous research indicates that MUC1 expression enhances the binding of the main salivary protein forming the MP, MUC5B. This study investigated the influence of MUC1 structure on MP formation. A TR146 cell line, which does not express MUC1 natively, was stably transfected with genes coding for three MUC1 isoforms differing in the structure of the two main extracellular domains: the VNTR domain, exhibiting a variable number of tandem repeats, and the SEA domain, maintaining the two bound subunits of MUC1. Semi-quantification of MUC1 using dot blot chemiluminescence showed comparable expression levels in all transfected cell lines. Semi-quantification of MUC5B by immunostaining after incubation with saliva revealed that MUC1 expression significantly increased MUC5B adsorption. Neither the VNTR domain nor the SEA domain was influenced MUC5B anchoring, suggesting the key role of the MUC1 N-terminal domain. AFM-IR nanospectroscopy revealed discernible shifts indicative of changes in the chemical properties at the cell surface due to the expression of the MUC1 isoform. Furthermore, the observed chemical shifts suggest the involvement of hydrophobic effects in the interaction between MUC1 and salivary proteins.

## 1. Introduction

The oral mucosa roughly consists of two main layers; the lower, inner lamina propria (made of connective tissue) and the surface stratified squamous epithelium. Oral epithelia can be classified into keratinized epithelium with a inflexible and tough surface, and non-keratinized epithelium with an elastic surface [1]. Keratinized epithelium is found on the hard palate and the gingiva, while non-keratinized epithelium can be found on lining mucosa, the soft palate, and buccal surfaces. Lining mucosa represents approximately 60% of the surface of oral mucosa [2]. Oral mucosae are subjected to numerous mechanical stresses (such as stretching, shearing, and friction forces), during orofacial movements, which include mastication, deglutition, speech, and oral care. To limit such friction strains, lubrication is ensured by a layer of salivary proteins called the mucosal pellicle (MP), tightly anchored to the epithelium, and a mobile salivary film. The MP is thought to contribute to moisture retention, lubrication, and protection against microbial colonization. It could also play a role in food flavour, the perception of astringency [3,4,5], and aroma persistence [6,7]. Lubrication is further ensured by a mobile salivary film (or residual fluid), defined as free saliva, which is retained on oral mucosa after deglutition. The thickness of the mobile salivary film varies, ranging from 70 to 100 μm [8] depending on the type of surfaces in the mouth [9]. Recent research reported that the salivary film thickness showed considerable differences based on its intra-oral location. For example, the salivary film at the anterior tongue and floor of the mouth was thickest, and the film at the anterior palate was thinnest. Similarly, MUC5B levels were highest at the anterior part of the tongue and lowest at the anterior palate [10].

The MP results from a specific anchoring of salivary proteins at the mucosal epithelial cell surface. The main salivary proteins forming the MP are the mucins MUC5B and MUC7 as well as secretory immunoglobulin A (sIgA) [11,12,13], together with low concentrations of numerous small proteins, such as secreted cystatins; histatins; and proline-rich proteins (PRP) [14]; and enzymes, such as amylases; carbonic anhydrase VI (CA VI); lysozyme; and glutathione transferases [15]. Currently, the mechanisms of MP formation and protein interactions are not fully understood. It has been proposed that hydrophobic effects play a prominent role in the adsorption of some salivary proteins at the surface of the epithelial cells [14]. Similarly, cross-linking between salivary proteins, facilitated by a catalytic reaction mediated by the enzyme transglutaminase (TGM), is involved in MP formation [16]. The adhesion of salivary proteins can also occur through protein–protein interactions. For instance, MUC7 and sIgA have been reported to form supramolecular complexes [17,18], and recent studies have shown that the expression of MUC1 by epithelial cells increases the anchoring of salivary proteins, especially MUC5B [19,20]. These results agree with hypotheses proposing that MUC1 plays a prominent role in salivary protein anchorage and MP formation [14,16,21]. However, information on the structural domains of MUC1 involved in the interaction is scarce. MUC1 is a dimeric transmembrane glycoprotein expressed by oral epithelial cells at their surface [22]. MUC1 consists of two subunits, α and β, linked together by non-covalent bonds located within a well-folded domain called the sea urchin sperm protein (SEA) domain [23,24,25]. The α subunit, which is exclusively extracellular, has a domain with a variable number of tandem repeats (VNTR domain) of a consensus sequence of 20 amino acids, ranging from 20 to 120 repeats in the northern European population [26,27]. This domain, rich in serine and threonine amino acids, is the site of extensive O-glycosylation, which contributes to the lubricating properties of MUC1 [28]. The negative charges of sialic acid residues at the extremity of O-glycosylation may also participate in the establishment of electrostatic interactions with salivary proteins. Thus, the aim of the present study was to investigate the impact of both the SEA and the VNTR domains of MUC1 on the anchoring of the salivary protein MUC5B and the formation of the MP.

The TR146 cell line, which does not express MUC1 [20], was stably transfected by three different isoforms of MUC1. These isoforms present a VNTR domain constituted of 20 repetitions, and they differ based on the structure of the SEA domain and the presence or not of enhanced green fluorescent protein (eGFP). The previously developed TR146-MUC1/Y-LSP cell line, which does not present the VNTR domain and has a truncated and non-cleavable SEA domain, was compared to these new cell lines.

## 2. Materials and Methods

### 2.1. MUC1 Gene Design and Construction

**Figure 1:**
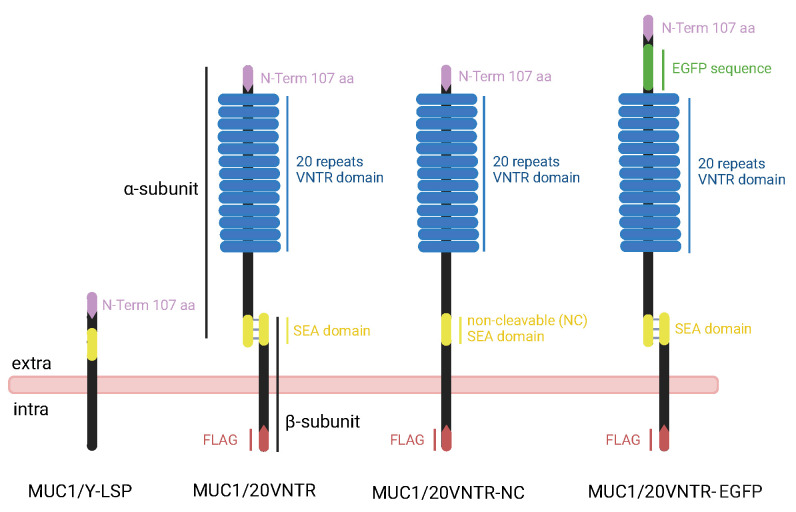
Schematic representation of the structure and major domains of the different isoforms encoded by the MUC1/20VNTR, MUC1/20VNTR-NC, and MUC1/20VNTR- EGFP constructs. Comparison with the structure of the truncated MUC1 isoform (MUC1/Y-LSP).

The amino acid sequence of human MUC1 was obtained from the online UniProtKB database (accession P15941) and used as a template to construct, in silico, 3 genes encoding different isoforms of MUC1. Figure 1 presents the structural differences between the different isoforms of MUC1. The first gene, named *MUC1/20VNTR*, encodes the MUC1 isoform 1, which consists of 2 subunits: the α-subunits, in the N-terminal domain, and β-subunit, in the C-terminal domain. The 2 subunits are connected at the level of the SEA domain and its autoproteolysis cleavage site. In addition, isoform 1 has a variable number of tandem repeats (VNTR domain) with 20 identical repeats of 20 amino acids (PDTRPAPGSTAPPAHGVTSA) on the α-subunit. The second gene, called *MUC1/20VNTR-NC*, encodes a second isoform, similar to isoform 1 but with the autoproteolysis cleavage site sequence of the SEA domain removed (NC). Finally, the third gene, *MUC1/20VNTR-EGFP*, encodes an isoform similar to isoform 1 but with an additional GFP sequence on the N-terminus upstream of the VNTR domain. To increase MUC1 protein expression, the Max sequence (QBI SP163 translational enhancer belonging to the pcDNA4/HisMax vector from Life Technologies Corporation (Thermo Fisher Scientic, Waltham, MA, USA) was introduced before the start codon. To allow detection and purification, the FLAG epitope tag (DYKDDDDK) was added to the C-terminus of each MUC1 construct. The 3 genes were synthesized commercially (Genewiz, Leipzig, Germany) and codon were optimized for mammalian cell expression. The synthetic cDNAs were subcloned into HindIII and NotI restriction sites of the pcDNA4 expression vector (Life Technologies, Waltham, MA, USA). The resulting expression plasmids, pcDNA4/Max-MUC1/20VNTR, pcDNA4/Max-MUC1/20VNTR-NC, and pcDNA4/Max-MUC1/20VNTR-EGFP, were transformed using electroporation and amplified in *Escherichia coli* ElectroMAX Stbl4 cells (Invitrogen, Waltham, MA, USA). Plasmid purifications were carried out using the QIAGEN Plasmid Plus Maxi kit (Qiagen, Courtaboeuf, France). To validate the sequence of amplified plasmid, Sanger sequencing (Genewiz) and alignments with the CLC Viewer software (CLC 23.0.5, QIAGEN Aarhus Prismet, Silkeborgvej 28000 Aarhus C Denmark) were performed to confirm the MUC1 isoforms’ sequences and differences.

### 2.2. Preparation of the Stable MUC1 TR146 Cell Lines

The human TR146 epithelial cell line, commonly used as a model of the buccal mucosa [29] (Rupniak et al., 1985), was obtained from the European Collection of Cell Cultures (ECACC, Salisbury, Wiltshire, UK). Cells were grown in DMEM/F12-GlutaMAX medium from Gibco (Carlsbad, CA, USA) (1:1, *v*/*v*) supplemented with 10% heat-inactivated foetal bovine serum (FBS), penicillin (100 units/mL), and streptomycin 100 µg/mL. The cells were cultured in T75 flasks and kept at 37 °C in a humidified atmosphere containing 7.5% CO_2_. The medium was changed every 2 days and cells were subcultured at confluency using Trypsin-EDTA (0.05%). The TR146/MUC1 cell line stably transfected with isoform Y-LSP, as previously described [5,6,19,20], was also used as a control for the cellular model of the oral epithelium. For the Y-LSP model, 2.5 mg/mL geneticin G418 (Gibco) was added to the culture media.

To perform transfection, TR146 cells were seeded at a concentration of 0.22 × 10^6^ cells/mL in T25 flasks and reached 80% confluence at 48 h. Then, the pcDNA4/Max-MUC1/20VNTR, pcDNA4/Max-MUC1/20VNTR-NC, and pcDNA4/Max-EGFP/MUC1-20VNTR-EGFP plasmids were linearised using 260 U of the restriction enzyme SSPI (stock solution: 10 U/µL (ThermoFisher, Waltham, MA, USA)), and individually transfected with Fugene HD (Promega, Charbonnieres Les Bains, France) with a reagent:DNA ratio of 3:1 (26 µL reagent:100 ng DNA) following the manufacturer’s instructions. After 24 h, transfected cells from each T25 were trypsinized, diluted, and seeded onto 100 mm cell culture dishes. After an additional 24 h of incubation, cells were subjected to antibiotic selection for 2–3 weeks using media supplemented with 25 µg/mL zeocin. The surviving cell colonies were subcloned using cloning rings. A total of 15 clones were expanded, stepwise, in 24-well plates, T25 flasks, and T75 flasks, and finally cryopreserved in liquid nitrogen (6 clones for isoform 1, 5 clones for isoform 2, and 4 clones for isoform 3). In parallel, each clone was screened to select the best stable cell line, according to 3 distinct criteria: the level of expression of MUC1, the addressing of the protein to the membrane, and its capacity to form the MP by anchoring MUC5B.

### 2.3. Saliva Collection and Application

Saliva was collected from 17 different subjects who reported to be in good oral health. Written informed consent was obtained from the participants. They were asked not to smoke, eat, or drink 2 h before the collection started. The collection of saliva lasted approximately 1 h during which subjects spat the saliva that naturally accumulated in their mouths into a plastic bottle without stimulation. All samples were then pooled and centrifuged for 20 min at 15,000× *g* and 4 °C to remove any food residue and bacteria. Aliquots of 4 mL of clarified saliva were prepared and frozen at −80 °C until use. When needed for cell treatment, the salivary sample was thawed on ice, diluted 1:1 in medium, and applied to cell culture for 2 h at 37 °C under humidified atmosphere containing 7.5% CO_2_ to create a salivary structure that mimics the oral MP. Then, the saliva solution was removed, and the cells were gently rinsed once with PBS to remove unbound proteins.

### 2.4. Fluorescent Immunostaining of MUC1, Plasma Membrane, and MUC5B

For immunostaining, TR146 (parental cells), TR146-MUC1/Y-LSP, and all TR146-MUC1 stable cell lines were seeded at 0.22 × 10^6^ cells/mL on 8-well culture plates (Corning Inc., Glendale, AZ, USA, 354108) and precoated with Corning Cell-Tak adhesive (Corning Inc., 354240) to reach 80–90% confluence in 48 h. For parental TR146 and all TR146-MUC1 stable cell lines, immunostaining of MUC1 was performed without a salivary pellicle, while immunostaining of MUC5B was performed with and without a salivary pellicle. To visualize the plasma membrane, cells were washed with pre-cooled PBS at 4 °C and incubated with biotin-conjugated concanavalin A, which binds to cell-surface glycoproteins, at 20 µg/mL (Merck, C2272-2MG) for 1 h at 4 °C. After PBS washing, cells were fixed using cold methanol–acetone (1:1, *v*/*v*) for a duration of 20 min, then fixed using cold 100% methanol for a duration of 20 min and washed again with PBS. To reduce non-specific binding, slides were blocked with 5% goat serum and 0.05% skimmed milk diluted in PBS for 1 h at room temperature (RT). For membrane staining, cells were incubated with Alexa Fluor 568 streptavidin conjugate (Life technologies, S11226) and diluted at 1:200 in PBS (final concentration 5 mM) for 1 h at RT. For MUC1 immunostaining and confocal analysis, we used the monoclonal mouse anti-FLAG M2 primary antibody (Sigma-Aldrich, Darmstadt, Germany, F1804) diluted at 1:250 in PBS (final concentration 4 mM) for 1 h at RT, followed by the secondary antibody Alexa Fluor 405 goat anti-mouse IgG (Life technologies, A48255) diluted at 1:2000 (final concentration 0.5 mM). For MUC5B immunodetection in epi-fluorescence, we used a mouse monoclonal anti-MUC5B antibody F2 [30] (Provided by Dr Floris Bikker, Free University of Amsterdam) diluted at 1:10 in PBS for 1 h, followed by the secondary antibody and then the Alexa Fluor 594 goat anti-mouse antibody (Life technologies, A32742) diluted at 1:400 in PBS (final concentration 2.5 mM). Finally, cells were washed 5 times with PBS, the chambers were removed from the slides, and the coverslips were placed with mounting medium Invitrogen ProLong Glass Antifade reagent with or without DAPI (Molecular Probes, Eugene, OR, USA, P36980 or P36935).

For MUC5B immunodetection, fluorescence was observed with a Nikon Eclipse E600 microscope (Nikon Instruments) equipped with UV-2A (excitation 330–380 nm, emission 400 nm) and G-2A (excitation 510–560 nm, emission 575 nm) filters. Images were acquired using a DS-Ri2 digital camera and analysed with the software NIS-Elements Basic Research (Version 4.30, Nikon Instruments, Melville, New York, NY, USA). For co-expression of MUC1 into the plasma membrane, images were acquired using a confocal laser scanning microscope (Leica TCS SP8) equipped with a 63 × objective lens (DimaCell platform, University of Burgundy, Dijon, France). Semi-quantification of MUC5B retained on the cell surface was performed using IgorPro 9 software (Version 9.05, WaveMetrics, Tigard, OR, USA) (*n* = 30 images for each MUC1 isoform), and statistical analysis was tested via Student’s *t*-test to validate differences between TR146 parental cells and TR146-MUC1 isoforms.

### 2.5. Semi-Quantification of the MUC1 via Dot Blot Analyses

For *MUC1* expression, each clone was amplified in T75 flasks and when cells reached 90% confluence, they were trypsinized, centrifuged at 1000× *g*, and the cell pellets were kept frozen at −80 °C until use. Cell pellets were lysed with an NP40 lysis buffer (Invitrogen) supplemented with mammalian protease inhibitor cocktail and RNase blocker (1/100th) solution. To improve protein extraction, the homogenate was further disrupted with a tissue lyser (TissueLyser, Qiagen, Hilden, Germany) and 3 mm tungsten beads (3 min agitation at 30 s^−1^ frequency), incubated for 1 h at 4 °C under strong shaking, and then centrifuged at 25,000× *g* for 20 min at 4 °C. Proteins were quantified in the resulting supernatant using the DC protein assay kit II (Bio-Rad, 5000112, Hercules, CA, USA). For the dot blot analysis, 3 replicates of lysate were produced and analysed for each clone. For each sample, 6 drops of lysate containing 3 µg of protein were deposited on 0.2 µm polyvinylidene difluoride (PVDF) membranes (Bio-Rad) and left to dry for around 30 min. Then, non-specific bindings were blocked in 0.3% skimmed milk. For MUC1 detection, we used the primary antibodies MUC1 mouse monoclonal antibody (OriGene Technologies, TA800838) or FLAG M2 mouse monoclonal antibody (Sigma, F1804), both diluted at 1:200 in PBS, and the secondary antibody goat anti-mouse IgG antibody HRP conjugated (Fisher, A24512), diluted 1:2000 in TBST. Enhanced chemiluminescence was used for the detection of HRP conjugates (1705061, BIO-RAD, 92430 Marnes-la-Coquette, France; BIO-RAD Clarity Western ECL Blotting kit used according to the manufacturer’s instructions). Images were acquired using the ChemiDoc MP Imaging System (Bio-Rad), with data acquisition via a camera (Bio-Rad), and data were analysed with ImageLab software (Version 3.0.1., Bio-Rad). The median values of technical replicate were calculated for each biological replicate and the difference between TR146 parental cells and TR146/MUC1 isoforms was evaluated via one-way ANOVA. When a significant difference was observed (*p* < 0.05), the means were compared using a Tukey pairwise comparison test (significance level set at 5%).

MUC5B immunostaining was observed with a NIKON Eclipse E600 microscope (Nikon Instruments, Melville, New York, NY, USA) after excitation with a high-pressure mercury-vapor lamp. Images were acquired using a Nikon Dxm1200C camera. The Nikon NIS-Elements BR software (Version 4.30) was used for data acquisition. Three images, taken of different areas of the sample, were acquired for each of the triplicates. Images of MUC5B immunostained samples were analysed using IgorPro 9 software (Version 9.05, WaveMetrics, Tigard, OR, USA). The sum of red layer intensity of pixels was determined for each picture. The effect of cell lines on MUC5B anchoring was evaluated via one-way ANOVA. When a significant difference was observed (*p* < 0.05), the means were compared using a Tukey pairwise comparison test (significance level set at 5%).

### 2.6. Immuno-Scanning Electron Microscopy (SEM) and Atomic Force Microscopy-Based Infrared Spectroscopy (AFM-IR)

Cells were seeded on 10 mm coverslips precoated with Corning Cell-Tak adhesive (354240 Corning Inc., Glendale, AZ, USA) and, as previously described, incubated with clarified saliva to reconstitute the MP. For SEM, cells were fixed for 30 min at RT with 4% paraformaldehyde diluted in PBS. After 2 washes, cells were incubated for 10 min in 50 mM NH_4_Cl diluted in PBS and washed again. Nonspecific binding sites were blocked with 0.3% skimmed milk and 5% goat serum for 15 min at RT. After 2 gentle washes, cells were incubated with FLAG M2 mouse antibody diluted at 1:25 in PBS for 1 h at RT, followed by incubation with a goat anti-mouse secondary antibody conjugated with 15 nm colloidal gold particles (Aurion, Wageningen, The Netherlands) diluted at 1:20 in PBS for 1 h at RT with shaking. The cells were fixed again with 1% glutaraldehyde for 5 min. Finally, the samples were dehydrated in successive ethanol baths of increasing concentrations ranging from 30% to 100% ethanol, for 20 min each. The samples were dried via critical point drying (CPD) using a Leica EM CPD030 (Leica, Wetzlar, Germany). Samples were coated with approximately 6 nm of carbon using a Quorum Q150T S Plus vacuum evaporator (QuorumTech, Lewes, UK) and observed with a Hitachi SU8230 scanning electron microscope equipped with a backscattered electron detector (Hitachi HighTech, 47807 Krefeld, Germany). The observations were carried out under an electron accelerating voltage of 5 kV at a working distance of 8 mm.

For AFM-IR analysis, cells were fixed with 1% glutaraldehyde for 5 min on CaF_2_ coverslips (Crystal GmbH, Berlin, Germany), followed by dehydration in successive ethanol baths of increasing concentrations ranging from 30% to 100% ethanol, for 20 min each.

The AFM-IR technique simultaneously provides nanoscale topography imaging offered by AFM, and chemical information retrieved via IR spectroscopy [31] in the range of 730 cm^−1^ to 1850 cm^−1^. As the applications of conventional infrared spectroscopy (IR) for nanoscopic analysis are constrained due to the low spatial-resolution performance set by the optical diffraction limit, AFM-IR achieves this goal by using the AFM probe to sense localised photothermal expansion induced by IR absorption [32]. This creates a force impulse on the tip of the cantilever causing the oscillation of the AFM cantilever probe [32,33]. By measuring the AFM probe oscillation to IR absorption, it is possible to acquire IR spectra at the nanoscale regions of the sample. In addition, by fixing the IR laser at specific wavelengths, AFM-IR measures absorption as a function of position across the sample by providing absorption maps which monitor nanoscale variation in chemical species accompanied by IR spectrum [34,35,36]. The nanospectroscopic characterization of TR146 and TR146-MUC1/20VNTR cell lines was conducted via nanoIR1, with excitation in ATR configuration using the CaF_2_ pyramid setup (Anasys Instruments Inc., Santa Barbara, CA, USA) in resonance-enhanced AFM-IR contact mode. The images were scanned utilizing HQ:CSC38/Cr Au probes with a stiffness of 0.07 N/m and scan rate of 0.4 Hz per line. The contact resonances were selected using a search location in the range of 170–190 kHz and a Gaussian filter with a width of 50 kHz. The maximum peak-to-peak amplitude of the oscillatory decay was used for recording AFM-IR spectra. The laser power was set at 15%.

## 3. Results

### 3.1. Expression and Localization of MUC1

#### 3.1.1. Expression of MUC1 in Stably Transfected TR146 Cells

**Figure 2:**
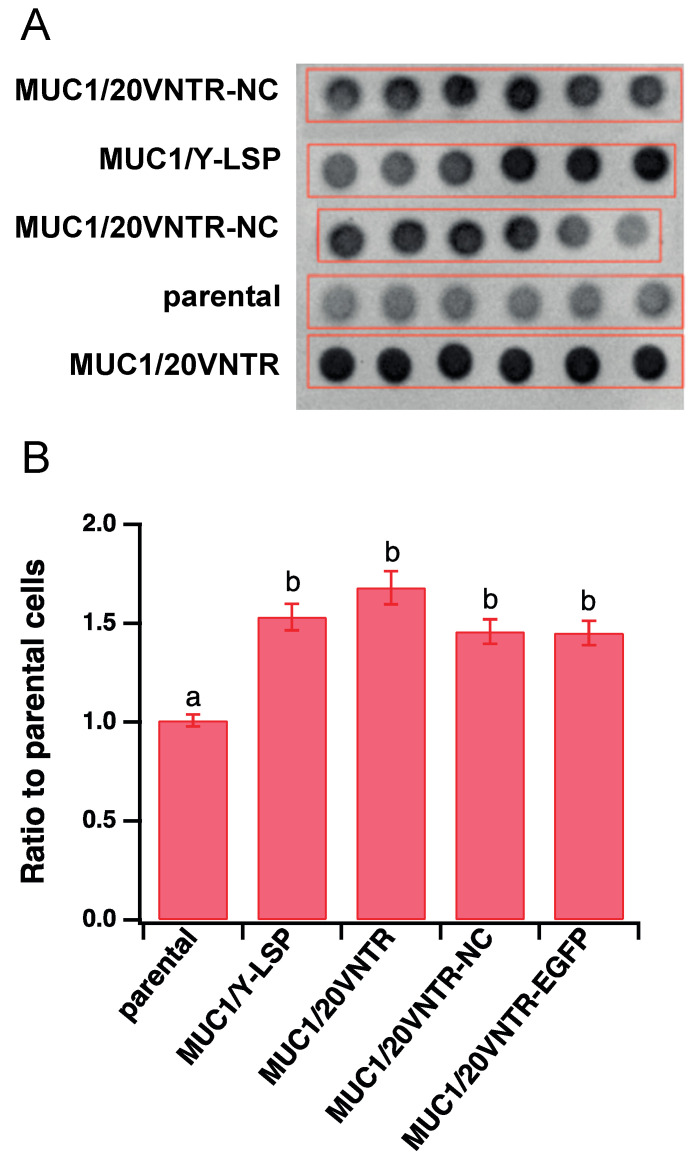
(**A**) Dot blot essay (hexaplicates) to detect the expression of MUC1 isoforms in transfected and un-transfected TR146 cells. (**B**) Semi-quantification (mean ± STD) of MUC1 isoforms expressed in the ratio of the background measured in TR146 parental cells (Different letters represent statistically significant differences; one-way ANOVA followed by Tukey pairwise comparison tests with *p*-value < 0.05).

Semi-quantification of MUC1 expression levels was performed via dot blot analysis (Figure 2), which revealed that anti-MUC1 antibody labelled MUC1 in cell extracts of transfected cell lines but not in parental TR146 cells. It also showed that the level of MUC1 expression was not significantly different between transfected cell lines.

#### 3.1.2. Cellular Localization of MUC1

**Figure 3:**
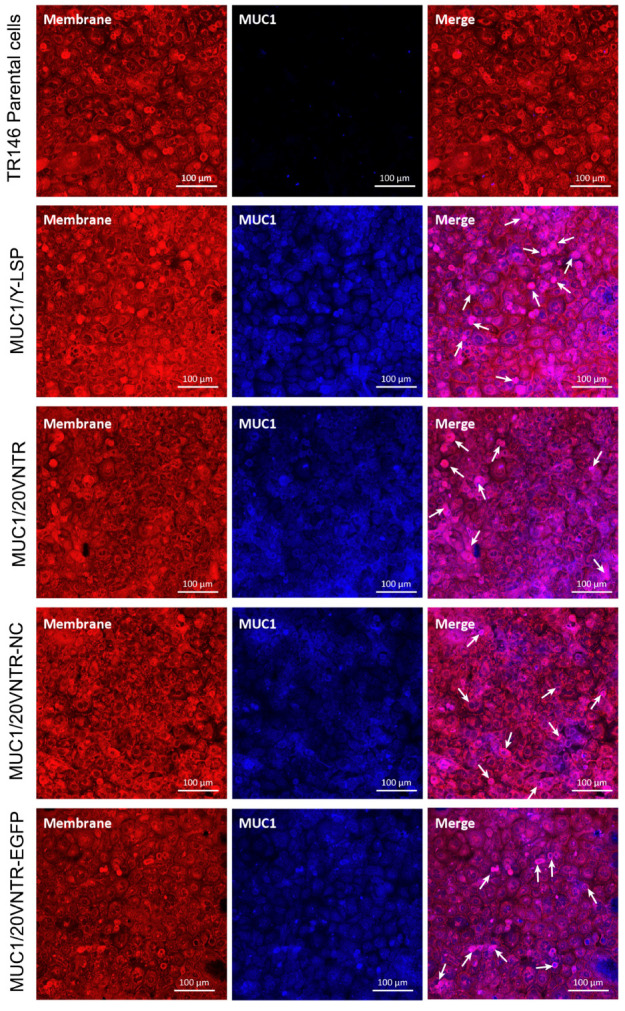
Co-immunostaining of the glycoprotein visualized using concanavalin A, a lectin known to selectively bind to cell-surface glycoproteins, depicted in red. Simultaneously, a secondary antibody labelled with Alexa Fluor 405, targeting the primary antibody anti-MUC1 (depicted in blue), is employed. The image was captured utilizing confocal microscopy. The regions of co-localization between the glycoprotein and MUC1 are represented by the magenta signal, highlighted by the white arrow.

To ensure proper localization of MUC1 to the cell membrane, fluorescent co-immunolabelling of the plasma membrane (red) and MUC1 (blue) was performed, followed by imaging with confocal microscopy (Figure 3). The left column of Figure 3 displays membrane localization in red, the middle column shows MUC1 localization in blue, and the right column illustrates the merged image for each TR146 cell line. No MUC1 expression was observed for parental cells, resulting in a red picture after merging. All transfected cell lines exhibited a distinct blue color in the middle column, indicating MUC1 expression, which seems to be in all parts of the cells. Merged images show a dominant co-fluorescence (magenta labelling), indicating that MUC1 and the cell membrane were colocalized. Thus, these images show that MUC1 was well expressed and addressed to the membrane in transfected cell lines.

### 3.2. Mucosal Pellicle (MP) Formation: Binding of the Salivary Protein MUC5B

**Figure 4:**
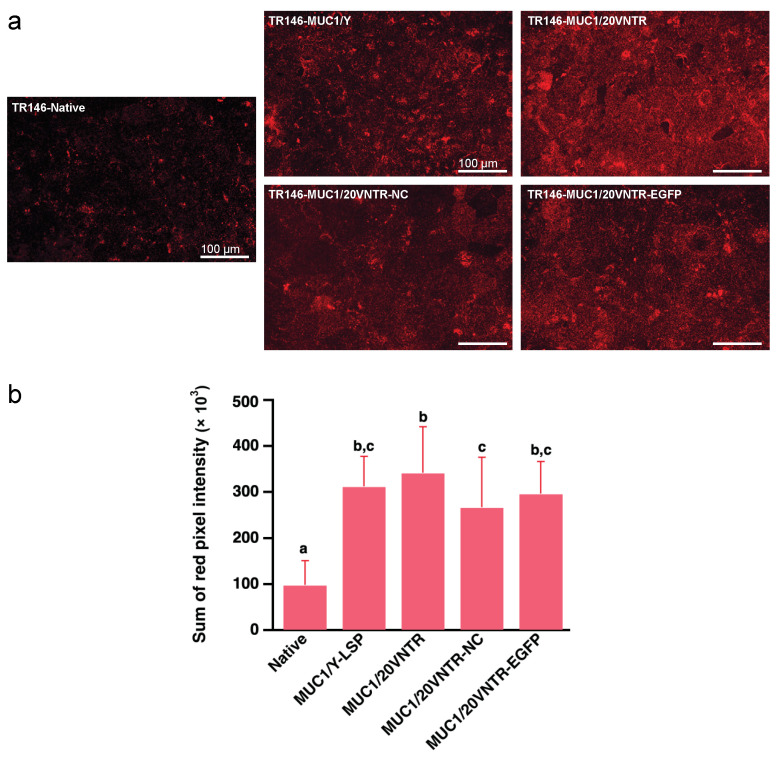
Anchoring of salivary mucin MUC5B to TR146 and TR146-MUC1 cells after incubation for 2 h with human saliva. (**a**) MUC5B immunostaining on TR146 and TR146-MUC1 cell surfaces, and (**b**) semi-quantification via image analysis (Different letters represent statistically significant differences; one-way ANOVA followed by Tukey pairwise comparison tests *n* = 45; *p*-value < 0.05). Scale bars 100 μm.

MP formation was evaluated by determining the amount of anchoring of the salivary protein MUC5B at the cell surface of the different cell lines. Figure 4a shows pictures of MUC5B immunostaining revealing the anchoring of MUC5B on the surface of the different cell lines. Image analysis of the fluorescence intensity revealed differences in MUC5B anchoring depending on cell lines. Significantly more MUC5B was anchored on the surface of transfected cells than on the surface of parental cells after 2 h incubation with human saliva (Figure 4a,b), while no significant differences were observed between the transfected cell lines. On average, the amount of MUC5B retained on the surface of MUC1-expressing cells was threefold higher than that on the surface of parental TR146 cells (*p* < 0.05).

**Figure 5:**
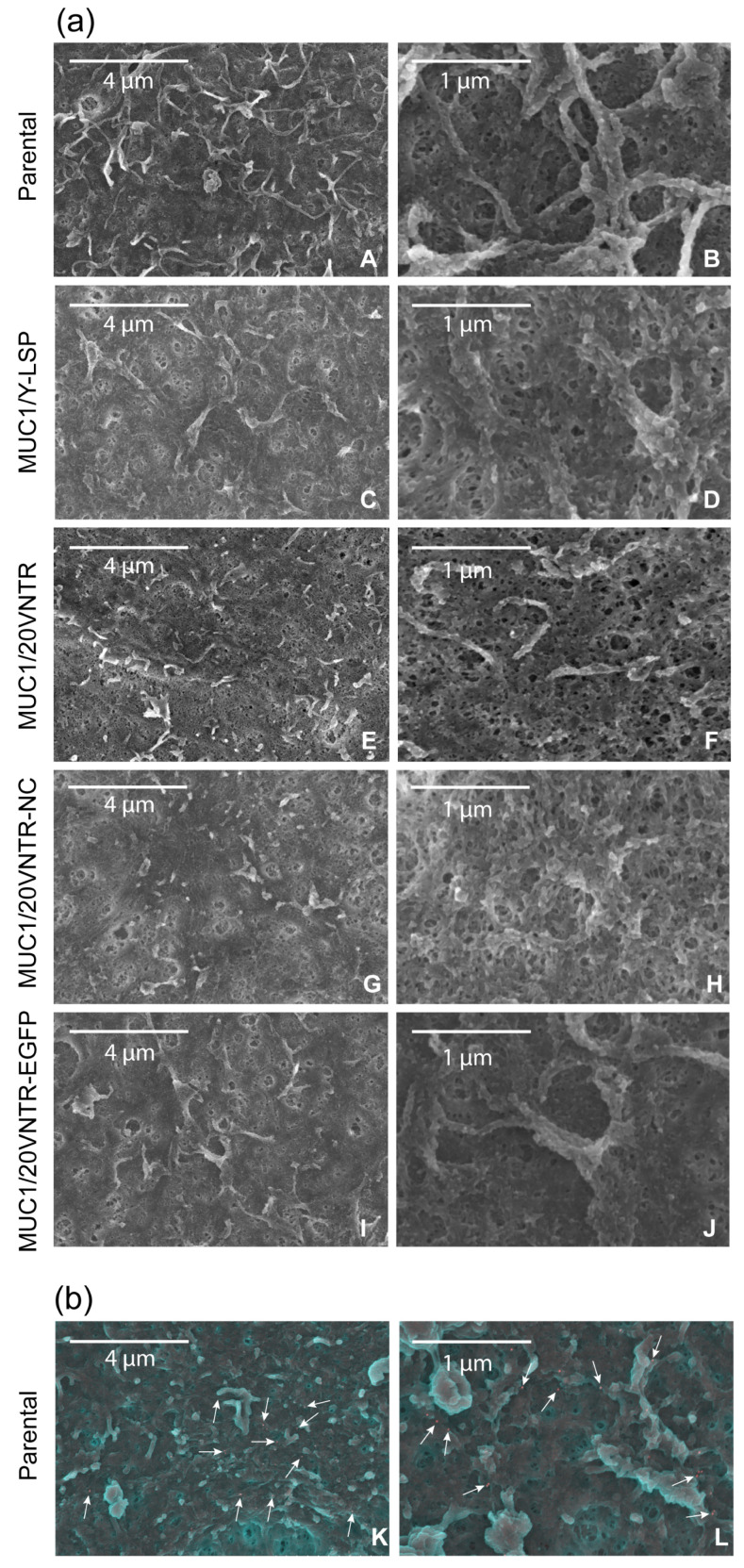
(**a**) SEM observation of TR146 (A,B) and TR146-MUC1 (C–J) cell surfaces after incubation for 2 h with clarified human saliva, and (**b**) (K,L) immuno-SEM detection of MUC5B salivary mucin (green) at the surface of TR146 cells. Gold particles are visible in red and indicated by white arrows. For (A,B) the scale bars are 4 µm, zoom ×12.0 K and 1 µm, zoom 45.0 K, respectively.

Scanning electron microscopy (SEM) was used to complement immunohistochemistry. SEM allowed observation of the structure of the mucosal film on the cell lines’ surfaces (Figure 5) and revealed that it consisted of a loose filamentous network. Slight differences could be observed between the parental cells (Figure 5a(A,B)) and cells transfected with MUC1 (Figure 5a(C–J)). Indeed, the structure of the microplicae could be clearly seen on the surface of the parental TR146 cells while it was less visible on the pictures of TR146/MUC1 cells, suggesting that the salivary deposit was thicker on the parental cells compared to TR146/MUC1. SEM coupled with MUC5B immunostaining was also performed (Figure 5b(K,L)) and confirmed the anchoring of MUC5B at the cell surface. These pictures also revealed few aggregates composed of MUC5B at the cell’s surface.

### 3.3. AFM-IR Nanospectroscopy Analysis of TR146 Buccal Cells: Impact of MUC1 Isoform on Chemical Properties of Cell Surfaces

AFM-IR was used to unravel the changes in the chemical and structural properties of TR146 buccal cells induced by the expression of the MUC1 isoform with 20 repeats of 20 amino acids on the α-subunit (MUC1/20VNTR). AFM-IR nanospectroscopy uses an AFM tip to sense nanoscale localized IR absorption and provides chemical information through IR spectra simultaneously with the nanoscale topography imaging.

**Figure 6:**
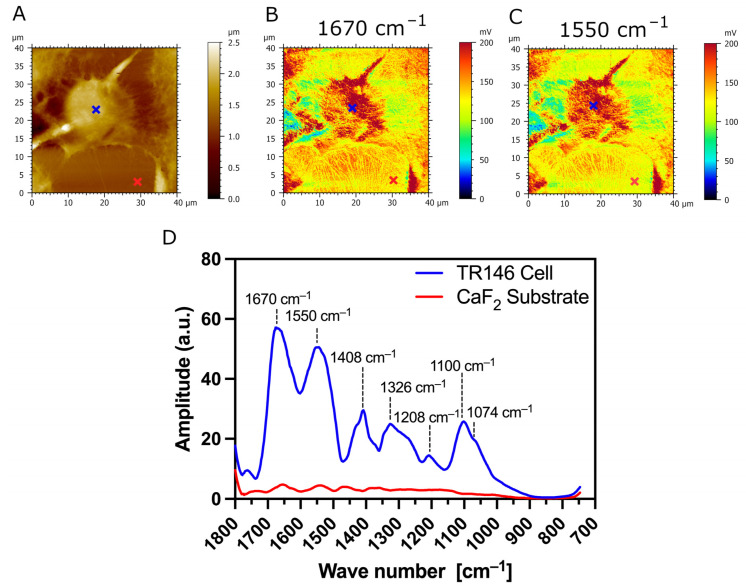
AFM-IR imaging of TR146 (parental) buccal cells. Size of imaged area: 40 × 40 µm. (**A**) AFM topography and (**B**,**C**) corresponding AFM-IR absorption maps of a parental TR146 cell at optimized wavenumbers 1670 cm^−1^ (amide I) and 1550 cm^−1^ (amide II), respectively. Both AFM-IR images show TR146 cells with higher IR absorption (intensive red color) compared to the underlying CaF_2_ substrate. (**D**) AFM-IR spectra were acquired on the cell’s surface (blue cross) and CaF_2_ (red cross). Both spectra were acquired at 1670 cm^−1^ and 1550 cm^−1^ and averaged and smoothed using a Savitzky–Golay filter (order 2, 10 pt). The prominent absorption bands in the ranges of 1600 cm^−1^–1800 cm^−1^, 1470 cm^−1^–1570 cm^−1^, and 1250 cm^−1^–1350 cm^−1^ are conventional protein bands associated with the amide I, amide II, and amide II bands, respectively. The absorption band with the peak at 1100 cm^−1^ is associated with carbohydrates.

The typical AFM topography image of parental TR146 buccal cells, and AFM-IR absorption maps with corresponding IR spectrum, are presented in Figure 6. Figure 6A presents the topography of the cells. Figure 6D presents AFM-IR spectra acquired on parental TR146 buccal cells and CaF_2_ substrate (blue and red annotations in Figure 6A–C). The two prominent absorption bands with peaks at 1675 cm^−1^ and 1548 cm^−1^ (see Figure 6D) are assigned to C=O stretching vibration (amide I band), and N-H bending vibration and C=N stretching vibration (amide II band), respectively [37]. Thus, the AFM-IR spectrum of parental TR146 cells shows conventional amide I and amide II protein bands. The absorption band in the range of 1356 cm^−1^–1466 cm^−1^ is a complex spectral region mainly associated with the symmetric stretching, deformation, and bending modes of the methyl groups (CH_3_) of proteins [38]. Within this absorption band, the peak at 1408 cm^−1^ is assigned to C-H bending and asymmetric vibrations of lipid acyl chains [39]. The low intensity peak at 1326 cm^−1^ in the range of 1234–1350 cm^−1^ is assigned to the amide III band overlapping with the shoulder at 1268 cm^−1^, which arises from CHα’ rocking vibration [38]. The absorption peak with the lowest intensity at 1208 cm^−1^ is assigned to phosphate bands [38]. The absorption fingerprint of carbohydrates of TR146 parental buccal cells is observed in the region of 1000–1164 cm^−1^ with a prominent peak at 1100 cm^−1^, assigned to stretching vibration of the C-O and C-C bands in the sugar ring [38]. Within this absorption band, the shoulder at 1074 cm^−1^ is associated with symmetric phosphate stretching modes [40]. Figure 6B,C present AFM-IR absorption maps at fixed wavenumbers 1670 cm^−1^ and 1550 cm^−1^ (Figure 6C), which correspond respectively to two major amide bands (amide I and II) [41]. The AFM-IR absorption maps of amides I and II show uniform protein concentration at the surface of parental TR146 buccal cells presented through the color code of AFM-IR absorption maps (intense red color in Figure 6B,C).

**Figure 7:**
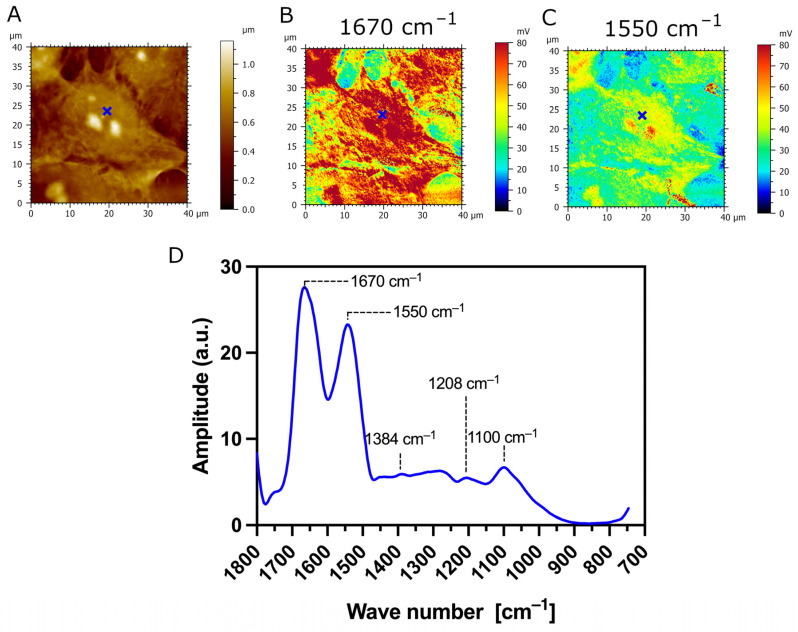
AFM-IR imaging of transfected TR146-MUC1/20VNTR buccal cells. Size of imaged area: 40 × 40 µm. (**A**) AFM topography and (**B**,**C**) corresponding AFM-IR absorption maps at optimized wavenumbers 1670 cm^−1^ (amide I) and 1550 cm^−1^ (amide II), respectively. Both AFM-IR images show transfected TR146-MUC1/20VNTR cells with higher IR absorption compared to the underlying CaF_2_ substrate. (**D**) AFM-IR spectrum acquired on the surface of TR146-MUC1/20VNTR (blue cross). This spectrum is the average of two spectra acquired at 1670 cm^−1^ and 1550 cm^−1^ on the cell’s surface and smoothed using a Savitzky–Golay filter (order 2, 10 pt). AFM-IR spectrum of TR146-MUC1/20VNTR shows prominent modification in the range of 1250–1350 cm^−1^ (amide III band) and 1358–1470 cm^−1^ (bending modes of methyl groups) compared to spectra acquired on TR146 (parental) cells.

The AFM-IR analysis of transfected TR146 cells which express the MUC1/20VNTR isoform is presented in Figure 7. The AFM-IR absorption maps scanned at 1670 cm^−1^ and 1550 cm^−1^ show IR absorption assigned to protein bands which are localized at the surface of transfected cells (intense red and yellow colors in Figure 7B,C, respectively). Figure 7D presents the AFM-IR spectrum acquired on transfected TR146-MUC1/20VNTR cells (blue annotations in Figure 7A–C). Two observed absorption bands with peaks at 1670 cm^−1^ and 1550 cm^−1^ (Figure 7D) exhibit strong IR absorbance assigned to the amide I and amide II bands, respectively. By comparing AFM-IR spectra acquired on parental TR146 cells (Figure 6D) and transfected TR146-MUC1/20VNTR cells (Figure 7D), we can conclude that absorption regions assigned to amide I and amide II bands remain intact. In addition, the AFM-IR spectrum acquired on TR146-MUC1/20VNTR shows an absorption peak at 1100 cm^−1^ and a band in the range of 1000 cm^−1^–1158 cm^−1^ assigned to the stretching vibrations of the C-O and C-C of polysaccharides [38]. Thus, the presence of the MUC1/20VNTR isoform does not impact the position and width of the absorption band associated with polysaccharides. In addition, the peak with the lowest intensity at 1206 cm^−1^, assigned to phosphate bands, is evenly presented in both AFM-IR spectra (Figure 6D and Figure 7D). However, it can be seen that the expression of the MUC1/20VNTR isoform by transfected cells particularly impacted the absorption bands in the region of 1238 cm^−1^–1468 cm^−1^, as presented in Figure 7D. In this region, a new absorption feature can be seen in the AFM-IR spectrum acquired on transfected TR146-MUC1/20VNTR cells (Figure 7D). By comparing AFM-IR spectra acquired on parental TR146 cells and TR146-MUC1/20VNTR cells (Figure 6D and Figure 7D), we can see that the peak at 1326 cm^−1^, assigned to C-N symmetry vibration (amide III band), is absent in the AFM-IR spectrum of transfected TR146-MUC1/20VNTR cells. The prominent absorption peak at 1408 cm^−1^ (Figure 6D) disappeared in the absorption band common in stretching, deformation and bending of CH_2_/CH_3_ groups (1362 cm^−1^–1468 cm^−1^). Instead, a peak with a low intensity is observed at 1384 cm^−1^, which is usually assigned to deformation of methyl groups of proteins (Figure 7D) [38,42]. The extracellular α subunit of MUC1/20VNTR isoform is extensively O-glycosylated and, besides the amino acid serine, it is constituted of threonine amino acids which possess methyl groups. Numerous conformational analyses and molecular modelling have reported that the structure of mucin-type glycopeptides strongly depends on the threonine amino acid involved in glycosylation [43,44,45]. In fact, the simple methyl group of threonine amino acids induces conformational limitation and a more rigid structure in Thr-linked glycopeptides compared to those attached only to serine amino acids [43]. Thus, the appearance of a low intensity peak at 1384 cm^−1^ on the AFM-IR spectra of TR146-MUC1/20VNTR cells may be due to threonine-linked O–glycopeptide residues, while the absence of absorption bands in the range of 1238 cm^−1^–1468 cm^−1^ (Figure 7D) could be induced by the limited conformational properties of Thr-linked glycopeptides.

**Figure 8:**
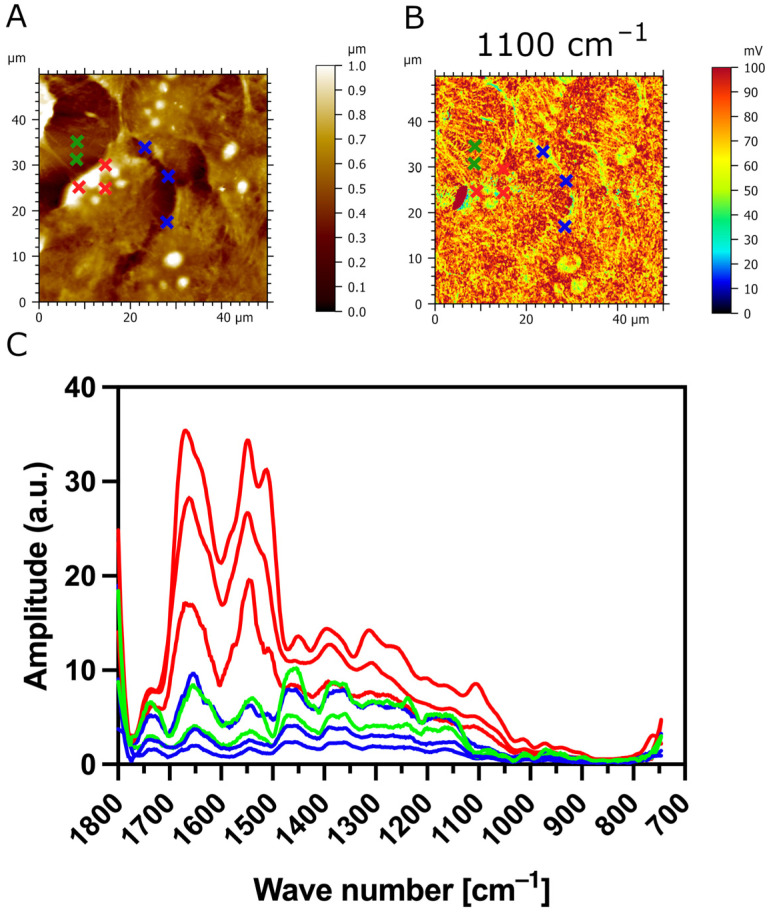
IR absorption measurement of TR146-MUC1/20VNTR as a function of wavelength at selected zones of interest. (**A**) AFM topography and (**B**) AFM-IR absorption map (1100 cm^−1^) of transfected TR146-MUC1/20VNTR cells with eight indicated areas of IR spectrum acquisition at three different zones: CaF_2_ substrate (green crosses), surface of TR146-MUC1/20VNTR cells (red crosses), and intercellular area (blue crosses). (**C**) The IR amplitude of the sugar absorption band (1000 cm^−1^–1158 cm^−1^) is prominently increased once the spectra are acquired on the cell’s surface (red lines) compared to IR spectra of CaF_2_ (green line) and intercellular spacing (blue line).

**Figure 9:**
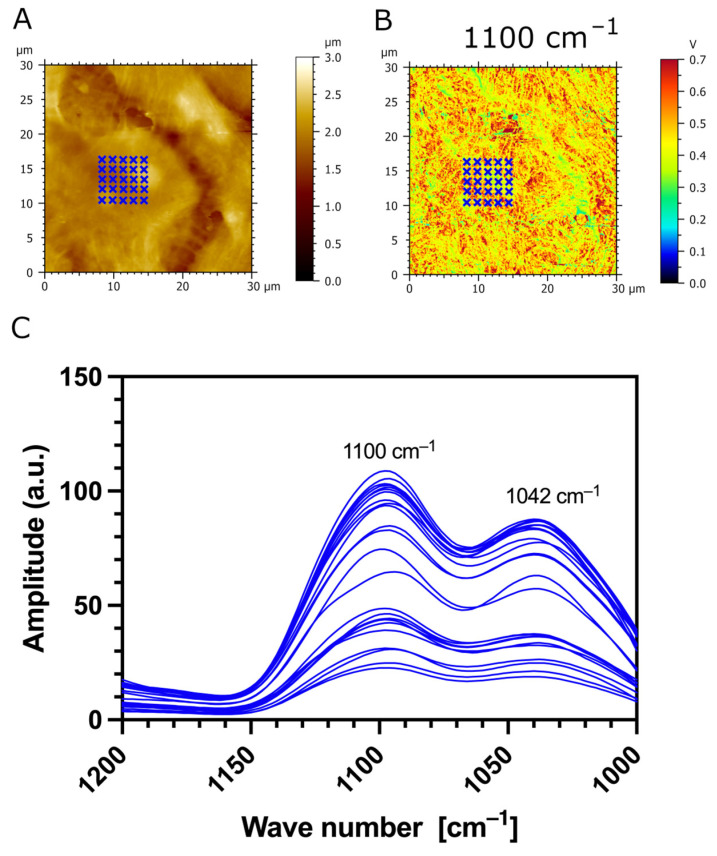
Measurement of IR absorption as a function of the restricted wavelength region assigned to sugar bands (1000 cm^−1^–1200 cm^−1^). (**A**) AFM topography and (**B**) AFM-IR absorption map (1100 cm^−1^) with 30 indicated zones (blue crosses) of IR spectrum acquisition on the surface of a TR146-MUC1/20VNTR cell. (**C**) IR spectra show two absorption peaks: 1100 cm^−1^, assigned to stretching vibrations of the C-O and C-C bands of polysaccharides, and 1042 cm^−1^, assigned to the glycogen band.

Additionally, we used AFM-IR nanospectroscopy to measure local IR absorption spectra (730 cm^−1^–1850 cm^−1^) at different zones of the samples. The IR spectra were acquired on the CaF_2_ substrate (outlined in green), the surface of transfected TR146-MUC1/20VNTR cells (outlined in red), and the cell’s spacing area (outlined in blue), as presented in Figure 8A,B. The most apparent increase in IR amplitude in amide absorption bands is observed in IR spectra acquired on the surface of TR146-MUC1/20VNTR cells (outlined in red). In addition, the IR amplitude of the absorption band assigned to polysaccharides (1000 cm^−1^–1158 cm^−1^) is noticeably increased in the acquisition zone on the cell’s surface (red lines), while this absorption band is not present in IR spectra acquired on CaF_2_ (green line) and intercellular spacing (blue lines). As MUC1/20VNTR is expressed on the surface of transfected cells, we conclude that the increase in the IR amplitude in the absorption region of 1000 cm^−1^–1158 cm^−1^ comes from the extensive O-glycosylation profile of MUC1/20VNTR [46,47]. Figure 9 presents IR absorption measurements acquired in a restricted wavelength range (1000 cm^−1^–1200 cm^−1^) on the surface of a TR146-MUC1/20VNTR cell; the appearance of an additional peak at 1042 cm^−1^ is visible (Figure 9C). This peak is assigned to the glycogen band (OH stretching), and it is usually referred to as a global estimation of glycosylation level [47].

## 4. Discussion

The present study aimed to evaluate the effects of the main structural domains of MUC1 on the formation of the MP on oral epithelial cells and, more generally, to obtain a deeper understanding of their involvement in the formation of the MP. Three new in vitro models of oral mucosa were proposed, based on the transfection of the TR146 cell line with three different isoforms of MUC1: MUC1/20VNTR, MUC1/20VNTR-NC, and MUC1/20VNTR-EGFP. Overall, MUC5B binding was quantitatively enhanced when oral epithelial cells expressed the different isoforms of MUC1. Any effect of the presence of the VNTR and the structure of the SEA domain was observed.

The new models were based on the transfection of the TR146 cell line, similar to the TR146-MUC1/Y-LSP cell line that was previously developed [20]. At their surfaces, TR146 cells exhibit the typical membrane folds of oral cells of non-keratinized surfaces, called microplicae (MPLs) [22]. MPLs are present at the surface of the TR146 cell line, as previously observed [20]. MPLs are typically present on the surfaces of areas covered with protective mucus. The function of MPLs is still not clear, but they may be involved in MP formation [48]. Additionally, MPLs increase the surface contact of the cells, favouring exchange with the environment. The presence of the transmembrane mucin MUC1 at MPLs’ apical end has been reported in vivo [22]. However, the TR146 cell line does not express MUC1 [22]. MUC1 is the only tethered mucin reported to play a role in the formation of the MP. As a membrane-bound protein, MUC1 appears to be a key protein in MP formation [20] because it provides an anchoring point for salivary proteins forming the MP, such as the gel-forming mucin MUC5B [49]. MUC1 is a highly glycosylated protein, participating in the lubrication of the oral mucosa by rendering its surface hydrophilic and electrostatic. These properties are probably also involved in the mucosa’s ability to interact with molecules, including flavour compounds [5,6,7]. Moreover, the expression of MUC1 has also been correlated with trigeminal sensitivity without a clear explanation [50].

MUC1 is a polymorphic protein with important interindividual variability at the sequence and glycosylation level. MUC1/1, the common isoform of MUC1, is composed of two noncovalently bound subunits: the α-subunit and the β-subunit. The α-subunit (N-terminal subunit) of MUC1/1 is entirely extracellular, forms a rigid structure that can extend 300–500 nm above the cell surface, and is composed of two main domains. The variable number tandem repeat (VNTR) domain consists of nearly identical repeats with abundant O-glycosylation on the serine and threonine residues. The VNTR domain comprises approximately 20–120 repeat units depending on the inherited polymorphism, with 40 and 80 repeats being the most frequent [51]. The SEA domain comprises approximatively 120 amino acids. It is a well-structured domain [24] that includes an autoproteolytic cleavage site. The β-subunit (C-terminal) comprises a short extracellular domain, a transmembrane domain, and a short cytoplasmic tail (CT), which has seven phosphorylation sites that support the role of MUC1 as a signalling molecule. MUC1/Y-LSP is differentiated from MUC1/1 by the absence of the VNTR domain and by a 15-amino-acid deletion “GVSFFFLSFHISNLQ” at the SEA domain N-terminus which, as a result, means it does not undergo cleavage. These two domains play an important role in the biological functions of MUC1. Glycosylation of the VNTR domain also participates in the formation of a physical barrier via steric hindrance [28,52,53] toward exogenous pathogens. The presence of the autoproteolytic cleavage site in the SEA domain allows the dissociation of the two subunits of MUC1 by mechanical action [24]. This mechanism may be part of a defence mechanism against pathogens or used to probe the extracellular environment. Microorganisms, which bind to the α-subunit, are eliminated by clearance after dissociation of the two subunits [54]. Similarly, the dissociation of the two subunits due to particular external conditions may act as a sensing function of MUC1 via the activation of intracellular signalling pathways [3,55]. However, there is no clue as to the role of these two domains in the formation of the MP and, in particular, the anchoring of MUC5B.

**Figure 10:**
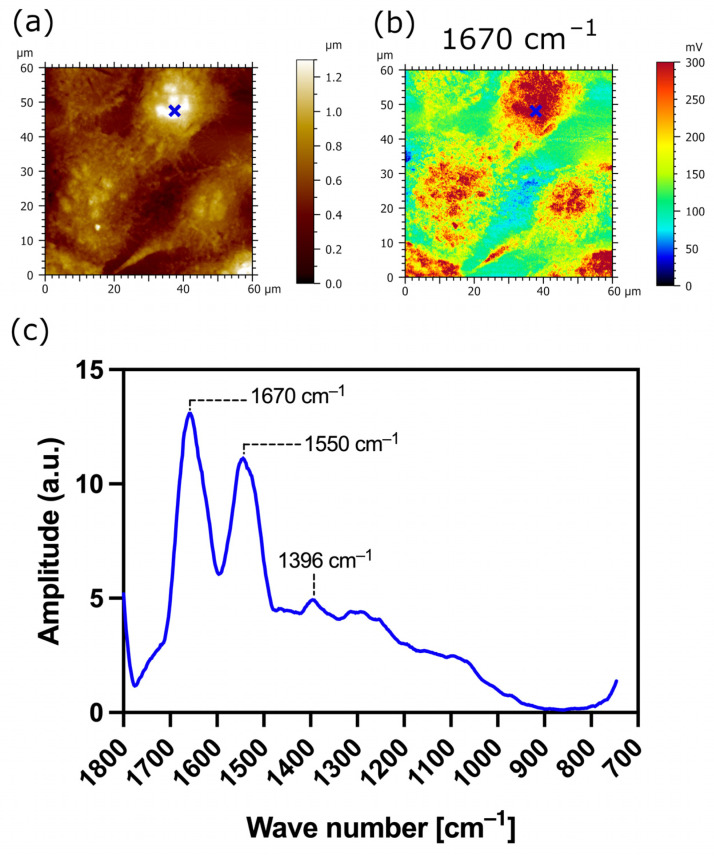
AFM-IR imaging of transfected TR146-MUC1/20VNTR buccal cells with saliva. Size of imaged area: 60 × 60 µm. (**a**) AFM topography and (**b**) corresponding AFM-IR absorption map at optimized wavenumber 1670 cm^−1^ (amide I band). (**c**) AFM-IR spectrum acquired on the surface of TR146-MUC1/20VNTR with saliva (blue cross). It shows an abortion peak at 1396 cm^−1^, which corresponds to symmetric CH3 bending of the methyl groups of proteins.

In order to unravel the structure-dependent function of the MUC1/20VNTR isoform, we performed AFM-IR nanospectroscopy on three cell lines: parental (TR146) cell lines, transfected TR146 cell lines without saliva (Figure 7), and transfected TR146 cell lines with saliva (Figure 10). Using the AFM-IR technique, we investigated the chemical properties of the transfected TR146 buccal cells induced by the MUC1/20VNTR protein (isoform 1), and the interactions involved in the anchoring of saliva on TR146-MUC1/20VNTR cells. The AFM-IR spectra of TR146-MUC1/20VNTR cell lines showed modification of the absorption bands in the region of 1238 cm^−1^–1468 cm^−1^ (Figure 7D) compared to the spectra of TR146 parental cells (Figure 6D). In this absorption domain, a low intensity peak was observed at 1384 cm^−1^, which is assigned to deformation of CH_2_ and CH_3_ groups [38,42]. This peak may have originated from threonine-linked O–glycopeptide residues of the MUC1/20VNTR isoform since threonine amino acids possess a methyl group. The simple methyl group of threonine amino acids influences conformational limitations in Thr-linked glycopeptides and, as such, could induce the appearance of a peak at 1384 cm^−1^ and the absence of absorption bands in the range of 1238 cm^−1^–1468 cm^−1^ (Figure 7D). We pursued AFM-IR investigation of TR146-MUC1/20VNTR cells with saliva (Figure 10). The AFM-IR spectrum (Figure 10c) revealed the absorption peak at 1396 cm^−1^, which is assigned to CH3 deformation bending of the methyl groups of proteins. The changes in the position of the CH3 absorption bands in the AFM-IR spectra in Figure 7D and Figure 10c (1384 cm^−1^–1396 cm^−1^) were induced by the presence of saliva and may have originated from hydrophobic interactions between the MUC1/20VNTR isoform and salivary proteins. Indeed, it has been reported that the hydrophobic interactions of the methyl groups of polypeptides cause a shift in C-H absorption bands to higher frequencies [56]. Thus, we can deduce that salivary proteins (MUC5B) are anchored at the surface of transfected TR126-MUC1/20VNTR cells by hydrophobic interactions established between methyl residues of the MUC1/20VNTR isoform and salivary proteins (MUC5B).

In this study, we confirmed that MUC1 expression at the cell surface increases the anchoring of MUC5B, while no significant effects of the VNTR or the SEA domain structures were observed. This suggests that these two structural domains are not involved in the anchoring of MUC5B. Another study reported biochemical interactions between the non-glycosylated CYS domains of MUC5B and the last 107 amino acids located at the N-terminus of MUC1 [57]. Thus, the last 107 amino acids of MUC1 may participate in the binding site of MUC5B. In agreement with this hypothesis, MUC5B has been reported to adsorb onto hydrophobic surfaces [14], possibly because the last 107 amino acids of the peptide chain represent a domain with hydrophobic properties. Thus, hydrophobic effects may be involved in the MUC5B anchoring on MUC1. At the opposite end, the hydrophilic properties of the VNTR may preclude the involvement of this structural domain in the interaction.

## 5. Conclusions

New in vitro models of oral mucosa were developed to investigate the role of the main structural domains of MUC1. These models confirmed that MUC1 is involved in the formation of the MP but suggest that the VNTR and SEA domain are not involved. Further investigations need to be conducted to confirm that the last N-terminal 107 amino acids of MUC1 comprise the binding site of MUC5B, participating in the formation of the MP. The MP is involved in many biological processes, such as surface lubrication; protection against bacterial colonization; and sensory perception, notably, aromatic persistence and astringency perception [5,6]. Thus, further studies should be performed to study the impact of the VNTR and SEA structural domains on the physicochemical properties of the cell lines and the ability of the MP to interact with flavour compounds. In view of this, we have recently put forward a new hypothesis of the molecular mechanisms underlying the sensory perception of astringency [3], in which MUC1 plays a key role, especially the SEA domain. The novel oral mucosa models developed in this study will allow us to investigate this hypothesis, and are poised to facilitate additional investigations aimed at advancing our comprehension of the role and structure of MUC1 in oral cavity lubrication, with potential implications for addressing issues related to oral dryness.

## Data Availability

The data presented in this study are available on request from the corresponding author.
